# Synthesis Optimization of BaGdF_5_:x%Tb^3+^ Nanophosphors for Tunable Particle Size

**DOI:** 10.3390/ma15238559

**Published:** 2022-12-01

**Authors:** Vladimir Polyakov, Zaira Gadzhimagomedova, Daria Kirsanova, Alexander Soldatov

**Affiliations:** The Smart Materials Research Institute, Southern Federal University, 344090 Rostov-on-Don, Russia

**Keywords:** X-ray photodynamic therapy, cancer treatment, BaGdF_5_ nanophosphors, terbium doping, tunable size

## Abstract

X-ray photodynamic therapy (XPDT) is aimed at the treatment of deep-located malignant tumors thanks to the high penetration depth of X-rays. In XPDT therapy, it is necessary to use materials that effectively absorb X-rays and convert them into visible radiation-nanophosphors. Rare-earth elements, fluorides, in particular, doped BaGdF_5_, are known to serve as efficient nanophosphor. On the other hand, the particle size of nanophosphors has a crucial impact on biodistribution, cell uptake, and cytotoxicity. In this work, we investigated various Tb:Gd ratios in the range from 0.1 to 0.5 and optimized the terbium content to achieve the maximum luminescence under X-ray excitation. The effect of temperature, composition of the ethylene glycol/water solvent, and the synthesis technique (solvothermal and microwave) on the size of the nanophosphors was explored. It was found that the synthesis techniques and the solvent composition had the greatest influence on the averaged particle size. By varying these two parameters, it is possible to tune the size of the nanophosphor particles, which make them suitable for biomedical applications.

## 1. Introduction

In terms of the number of deaths, malignant tumors rank second among all diseases and are one of the most difficult problems in health care [1,2,3]. Currently, there is no universal way to cure them without causing severe damage to the body. Existing methods of radiotherapy and chemotherapy, in addition to destroying malignant cells, also destroy healthy cells. Thus, the need to develop alternative and more safety treatment methods is still an important issue. One of these new non-invasive and low-toxicity methods is photodynamic therapy (PDT). The principle action of drugs for PDT is based on the suppression tumor growth by the generation of reactive oxygen species (ROS) [4,5] upon irradiation of a photosensitive substance (photosensitizer) [6,7,8]. However, the low penetrating power (<1 cm) of near-IR- or UV-radiation imposes significant restrictions on the use of PDT. Tumors that grow deep in the body remain inaccessible. X-rays can be used to increase the depth of penetration, which is a crucial benefit of X-ray photodynamic therapy (XPDT) [9,10]. However, it is necessary to introduce an additional component into the “photosensitizer-ROS” system, which would absorb X-rays and re-emit them into UV–Visible luminescence [11]. One of the most promising and studied are X-ray phosphors based on NaGdF_4_ and BaGdF_5_ doped with Eu^3+^, Tb^3+^, or other lanthanides [12,13,14,15,16,17,18]. Its advantages include high chemical stability, resistance to X-ray, and photochemical degradation as well as the ability to fine-tune multicolor luminescence by varying the type and concentration of the dopant ions [19,20,21,22,23,24,25,26,27,28]. Furthermore, materials based on BaGdF_5_ have been relatively poorly studied, and therefore are of greater interest to researchers. On the other hand, the replacement of Na by heavy Ba in the structure leads to an increase in the content of heavy metal atoms per formula unit without a significant increase in toxicity, which improves the X-ray attenuation capability, thus making it more suitable for X-ray micro-CT.

To obtain the maximum luminescence yield, it is necessary to correctly select the concentration of the dopant. Weak luminescence can be caused either by a too low dopant concentration or by the process of quenching the luminescence at a high concentration [29,30,31,32]. Another important criterion for the applicability of composites is their high biocompatibility.

The particle size plays an important role. Too small particles can be quickly filtered out by the body’s defense systems without accumulating in the tumor. On the other hand, too large particles are not able to penetrate the biomembranes and accumulate only at the site of their introduction without subsequent penetration into the cells. Nanoparticles (NPs) with a particle size in the range of 50–200 nm are considered optimal for biomedical applications [33]. However, the size of smaller particles (10–15 nm) can be increased by coating them with biocompatible shells such as silica [34,35,36,37]. In this case, the porous structure of silica makes it possible to adsorb the photosensitizer molecules.

The size of the resulting nanoparticles can be controlled by varying synthesis parameters such as precursor concentration [38,39], temperature [40,41], choice of solvent [42], reaction time [20], and synthesis method. Temperature variations can affect the rate of reactions and consequently the rate of crystallite growth. The choice of solvent also plays an important role. Recently, the synthesis of nanoparticles in organic solvents, for example, polyols, has become increasingly popular [42,43]. The most optimal solvent in BaGdF_5_ synthesis is ethylene glycol (EG). Ethylene glycol acts not only as a solvent, but also as a surfactant. Being adsorbed on the surface, it is able to block the growth of the particles as well as prevent their agglomeration. The addition of water to ethylene glycol can strongly influence the growth of crystallites. This occurs due to the competition of solvents in the coordination sphere of metal ions. Water molecules are a more preferable ligand; therefore, they actively influence the adsorption of ethylene glycol on the surface of the formed particles [44,45]. Techniques for obtaining BaGdF_5_ nanoparticles of various sizes using ionic liquids (IL) as structure-driven agents are also known [20,38]. An ionic liquid is a salt in a liquid state. Ionic liquids are environmentally friendly solvents, but they can also act as reagents. For example, IL based on tetrafluoroborates [BF_4_]^−^ can themselves be a source of fluoride ions, which makes it possible to simplify the synthesis process.

To obtain BaGdF_5_, solvothermal synthesis is usually used. This technique is well-studied and has good reproducibility. However, the long synthesis time (24 h) is its drawback. We have developed a microwave synthesis technique, which makes it possible to reduce the synthesis time by 10 times [46]. Another important advantage of microwave synthesis over hydrothermal synthesis is not only the reaction rate, but also the uniformity of the reaction volume heating. This allows for the homogenization of the direction of the chemical reactions throughout the volume.

Previously, we studied BaGdF_5_ nanoparticles doped with Eu^3+^, obtained by microwave synthesis, coated with silica, and impregnated with methylene blue as a photosensitizer [37]. In this paper, we optimized the Tb^3+^ content to obtain the maximum luminescence yield. We also studied how microwave and hydrothermal techniques, temperature, and various ratios of EG/water affect the particle sizes of the synthesized nanophosphors.

## 2. Materials and Methods

### 2.1. Materials

GdCl_3_, TbCl_3_ ∙ 6H_2_O, BaCl_2_ ∙ 2H_2_O, ethylene glycol, polyethylene glycol (PEG, M = 1500 g/mol), and NH_4_F were purchased from Sigma-Aldrich Co. (St Louis, MO, USA).

### 2.2. Methods

Samples were prepared using a high-temperature autoclave as well as in a microwave CEM Mars6 reactor (CEM Corporation, Matthews, NC, USA). X-ray powder diffraction (XRD) was measured by means of Bruker D2 PHASER using Cu Kα radiation (λ = 1.5406 Å) at 30 kV, 10 mA, and the following parameters: 2θ range from 5 to 90, step size of 0.01. An FEI Tecnai G2 Spirit BioTWIN was used to perform TEM for imaging of the obtained samples. An accelerating voltage of 80 kV was used. The elemental composition was analyzed using micro-X-ray fluorescence spectrometer M4 TORNADO (Bruker, Billerica, MA, USA). The X-ray excited optical luminescence (XEOL) signal was detected by using an Agilent Cary Eclipse fluorescence spectrophotometer with the emission slit set to 10 nm and the following parameters of the X-ray tube: voltage 35 kV and current 1.6 mA. Powder samples were deposited on the thin film, which was fixed in a way that results in an angle of 45° between the sample surface and both the X-ray beam and fluorescence detector window.

### 2.3. Synthesis

#### 2.3.1. Terbium Content Optimization

To optimize the terbium content, microwave synthesis developed by our scientific group was used. According to the procedure [46], samples were prepared with a terbium content of 5, 10, 25, and 50%. Briefly, a 20 mL solution of a barium, gadolinium, and terbium chloride mixture in ethylene glycol (EG) was prepared using sonication (see Table 1). Then, 1.5 g of polyethylene glycol (PEG-1500) was added to the solution and again sonicated until complete dissolution. After that, 10 mL of ammonium fluoride solution in ethylene glycol was added to the chloride solution.

The resulting mixture was transferred into Teflon ampoules and placed in a CEM Mars6 microwave reactor. The synthesis conditions were as follows: temperature—200 °C, heating time—2 h. After that, the mixture was washed by centrifugation (13,000× *g* rpm for 10 min) three times with water. The resulting solution was dried at 60 °C.

#### 2.3.2. Variation of Synthesis Conditions

The synthesis conditions were varied to obtain particles of the maximum size. Variable parameters included the type of synthesis (microwave MW or solvothermal ST), solvent composition (ethylene glycol/water), and temperature. The detailed synthesis conditions are presented in Table 2.

## 3. Results and Discussion

### 3.1. Terbium Content Optimization

#### 3.1.1. X-Ray Diffraction of BaGdF_5_: x%Tb^3+^

**Figure 1:**
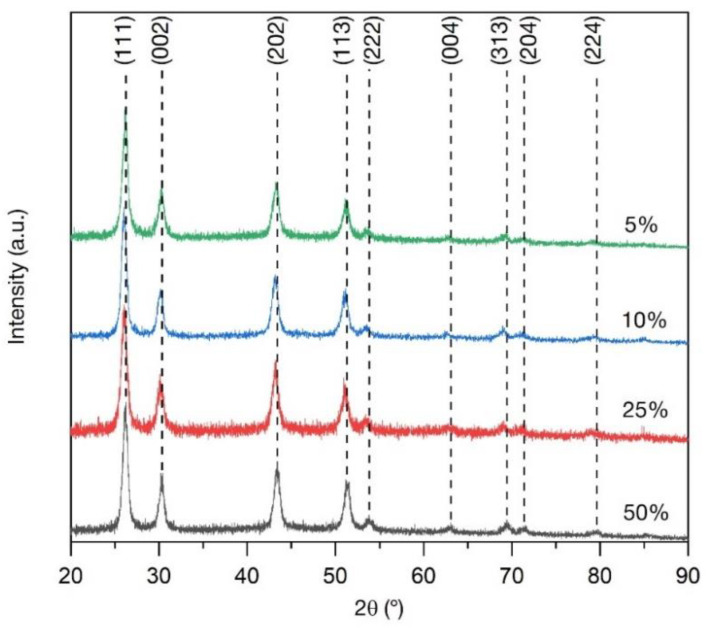
XRD patterns of the BaGdF_5_: x%Tb^3+^ samples synthesized by the MW method (where x reported the legend).

The profile analysis was carried out using the Jana2006 program package (version 25/10/2015) [47]. In our previous work [16], it was stated that all synthesized samples crystallized in the single cubic space group Fm-3m (225) (JCPDS card no. 24-0098 [48]). The XRD patterns are shown in Figure 1.

**Figure 2:**
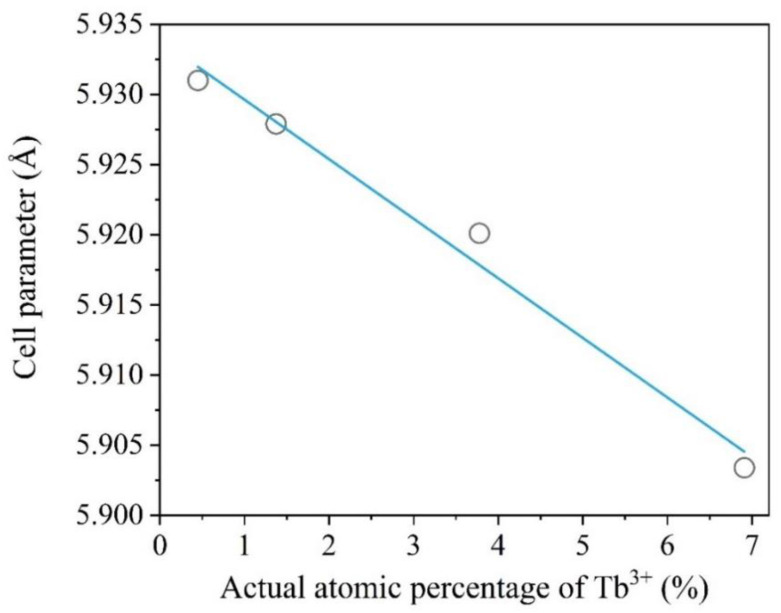
Correlation between the actual percentage of doping Tb^3+^ ions and the refined lattice parameter.

The refined (Figures S1 and S2) value of the cell parameters and cell volumes are presented in Table 3 and Figure 2. The profile parameters were refined using the pseudo-Voigt function.

According to the refined values, it can be observed that the doping of the BaGdF_5_ crystal structure with Tb^3+^ ions led to the decrease in the cell parameters. This fact can be explained by the analysis of the ionic radius of the doping element. The ionic radii of the hexacoordinated Gd^3+^ and Tb^3+^ were 1.078 and 1.063 Å, respectively [49]. Thus, it can be seen that the Tb^3+^ ions had a smaller ionic radius and, therefore, the increase in the dopant content led to a decrease in thee cell parameters (Figure 2). The observed trend confirmed that Tb^3+^ substitutes Gd^3+^ in their lattice positions. The presence of possible side-phases was excluded based on the XRD and XRF data.

Moreover, the Scherrer equation was used for the estimation of the average crystalline size. In this case, shape factor k was chosen for spherical particles (k = 0.9) because according to the TEM data, synthesized nanoparticles are close to spherical shape (see Section 3.1.3). The analysis of the full width at half-maximum (FWHM) of the XRD lines showed that for the synthesized 5Tb MW, 10Tb MW, 25Tb MW, and 50Tb MW nanoparticles, the averaged sizes were equal to 8.5, 8.9, 7.7, and 10.3 nm, respectively. The obtained results were in good correspondence to thee averaged particle size evaluated explicitly from the TEM images.

#### 3.1.2. X-Ray Fluorescence Measurements of BaGdF_5_: x%Tb^3+^

The elemental composition of the BaGdF_5_ doped with different initial concentrations (x = 5, 10, 25, 50%) of Tb^3+^ was estimated using X-ray fluorescence (XRF) data. The actual and expected elemental composition percentage of the Ba, Gd, Tb, and F elements for each sample were calculated and reported in Table S1. The obtained results demonstrate that the Tb^3+^ precursor almost entirely reacted during the synthesis involved in the formation of NPs and that BaGd_1–x_F_5_Tb_x_ stoichiometry is well-established. Some of the notable deviations between the predicted and actual Tb^3+^ content observed for the lowest Tb^3+^ loading were likely due to the presence of a tiny amount of the nonreacted Tb^3+^ precursor as well as an XRF experimental error in determination at a low-concentration level.

#### 3.1.3. Transmission Electron Microscopy (TEM) of BaGdF_5_: x%Tb^3+^

**Figure 3:**
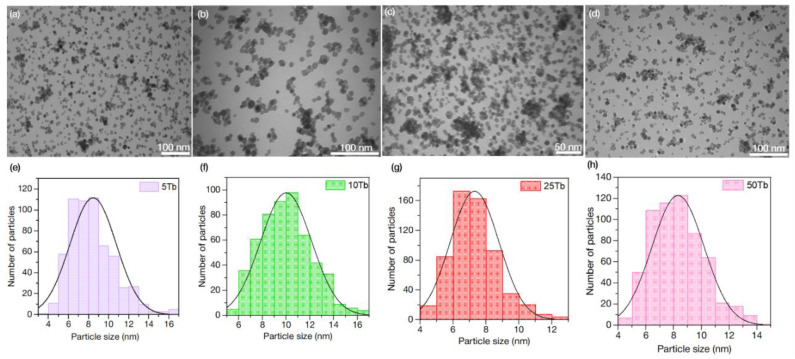
TEM images of (**a**) 5Tb MW, (**b**) 10Tb MW, (**c**) 25Tb MW, and (**d**) 50Tb MW; Particle size distribution of (**e**) 5Tb MW, (**f**) 10Tb MW, (**g**) 25Tb MW, and (**h**) 50Tb MW according to the TEM analysis.

The size distribution of the nanoparticles was estimated using the ImageJ program [50] by analyzing the TEM images (Figure 3). The total numbers of the measured nanoparticles were about 600. As a result, it has been shown that for all samples, the nanoparticles were in the range of 5–17 nm. This fact complies with the requirements for nanophosphors as a part of nanocomposites that can be used in XPDT. Moreover, the TEM results are in good agreement with the crystalline size according to the Scherrer analysis (see Section 3.1.1).

#### 3.1.4. X-Ray Excited Optical Luminescence

It is widely known that rare-earth metal ions such as Tb^3+^ doped into a wide range of materials exhibit optical luminescence upon UV–Vis and X-ray radiation [14,28,51,52]. The latter makes it possible to convert ionizing radiation into visible light as part of the XPDT system.

**Figure 4:**
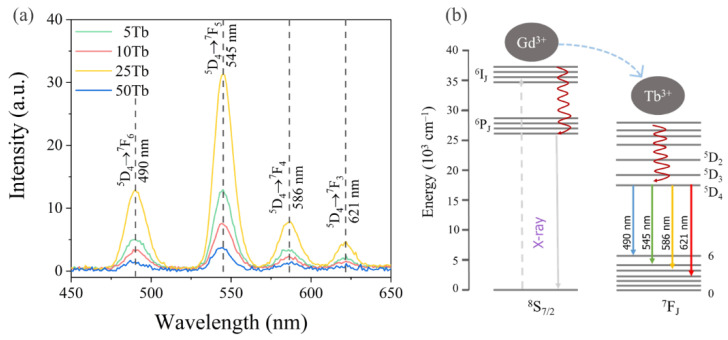
(**a**) X-ray-excited (U = 35 kV, I = 1.6 mA) optical luminescence spectra measured for x% of Tb^3+^; (**b**) schematic energy-level diagram showing the processes occurring in BaGdF_5_:Tb^3+^.

The XEOL spectrum (Figure 4) of samples doped with x%Tb showed a spectral shape typical for Tb^3+^-ions doped into the BaGdF_5_ structure [14,28]. According to the emission spectrum of x%Tb^3+^, four strong narrow bands at 490, 545, 586, and 621 nm were ascribed to the electronic transitions from the excited state of ^5^D_4_ to the ground states of ^7^F_J_ (J = 6–3) [53], as shown in Figure 4b: ^5^D_4_ → ^7^F_6_ for λ = 490 nm, ^5^D_4_ → ^7^F_5_ for λ = 545 nm, ^5^D_4_ → ^7^F_4_ for λ = 586 nm, and ^5^D_3_ → ^7^F_3_ for λ = 621 nm. The dominant peak was the green emission of 544 nm, which is a magnetic dipole transition with ΔJ = 1, and was more intense than the other transitions [52,53,54]. As shown in Figure 4a, we may declare that the optimal actual content of doped Tb^3+^ was equal to 3.78 at. % (i.e., 25Tb). Thus, the 25Tb structure was chosen for further synthesis improvements.

### 3.2. Tuning Size of BaGdF_5_: 25Tb^3+^ Nanoparticles by Various Synthesis Conditions

#### 3.2.1. X-Ray Diffraction of BaGdF_5_: 25Tb^3+^ Obtained by the MW and ST Techniques

**Figure 5:**
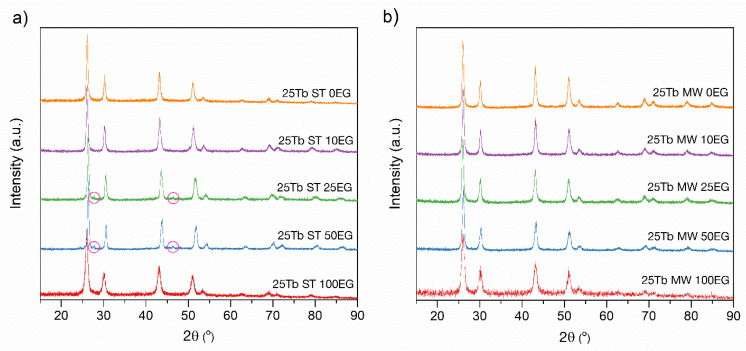
XRD patterns of the BaGdF_5_: 25%Tb^3+^ prepared with (**a**) ST and (**b**) MW synthesis. The impurities of the GdF_3_ phase marked with pink circles.

The XRD patterns of all MW and ST samples are presented in Figure 5. It can be seen that for solvothermal synthesis in a system containing only ethylene glycol (25Tb ST 100EG) as a solvent, the rate of crystal nucleation was low due to the high viscosity and slow diffusion of the reagents. The system had enough time to heat up before the formation of the crystals started, thus the pure BaGdF_5_ phase was formed.

However, the viscosity of the solution dropped, and the reaction rate increased with the addition of water to the system (25Tb ST 25EG and 25Tb ST 50EG). Since there were many more Ba^2+^ and Gd^3+^ ions in the system than Tb^3+^ ions, the statistical probability of the predominant formation of BaF_2_, BaGdF_5_, and GdF_3_ in the first stage was higher than that of their terbium analogues. Then, after the formation of the BaGdF_5_ and GdF_3_ particles, gadolinium was replaced by terbium. Moreover, based on the standard dissociation energies of the Gd–F bonds (590 kJ/mol [55] and Tb-F (561 kJ/mol [55]), it can be seen that the process of replacing Gd^3+^ with Tb^3+^ ions is endothermic and therefore proceeds at an elevated temperature. It can be assumed that in the case of hydrothermal synthesis, the heating of the reaction mixture occurs unevenly. These factors, in combination with the slow diffusion of reagents, in particular terbium ions, lead to the fact that the substitution process proceeds more actively in the solvent layer located near the hot walls of the ampoule than in the deep layers. As a result, the small impurities of the GdF_3_ phase [56] in the diffraction profiles of these samples can be seen (marked with pink circle at Figure 5a).

In addition, an excess of water in the system (25Tb ST 0EG and 25Tb ST 10EG) led to an even greater drop in the viscosity and facilitated the diffusion of reagents. On the other hand, the thermal conductivity of the solution increased from 0.249 W/m·K for pure ethylene glycol [57] to 0.569 W/m·K for pure water [57]. These factors caused a more uniform reaction over the volume. As a result, the pure BaGdF_5_ phase was observed from the XRD (Figure 5a,b).

#### 3.2.2. Transmission Electron Microscopy Measurements of BaGdF_5_:25Tb^3+^ Obtained by MW and ST Techniques

**Figure 6:**
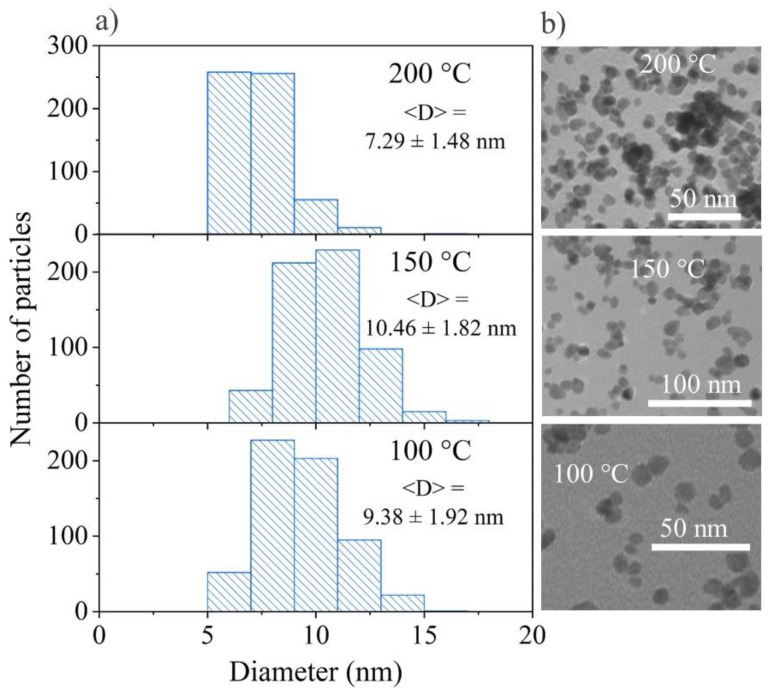
(**a**) Particle size distribution and <D> with the mean size ± standard deviation; (**b**) TEM images of 25Tb MW at 100 °C, 150 °C, and 200 °C.

**Figure 7:**
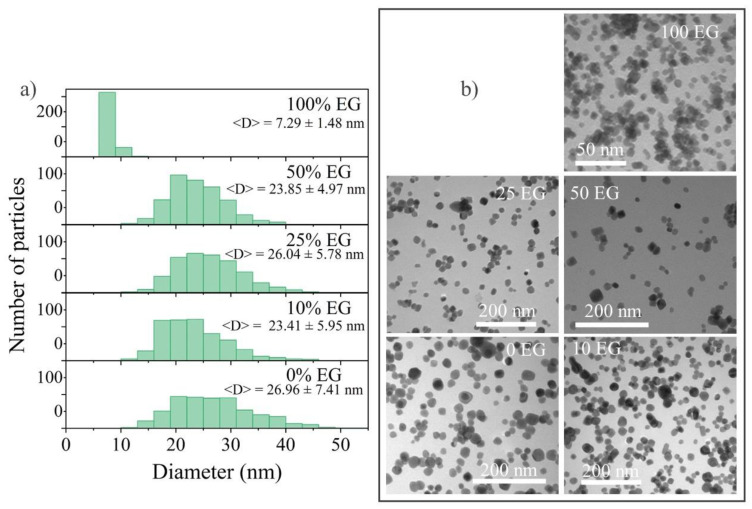
(**a**) Particle size distribution and <D> with the mean size ± standard deviation; (**b**) TEM images of 25Tb MW with different contents of EG 0, 10, 25, and 50%.

**Figure 8:**
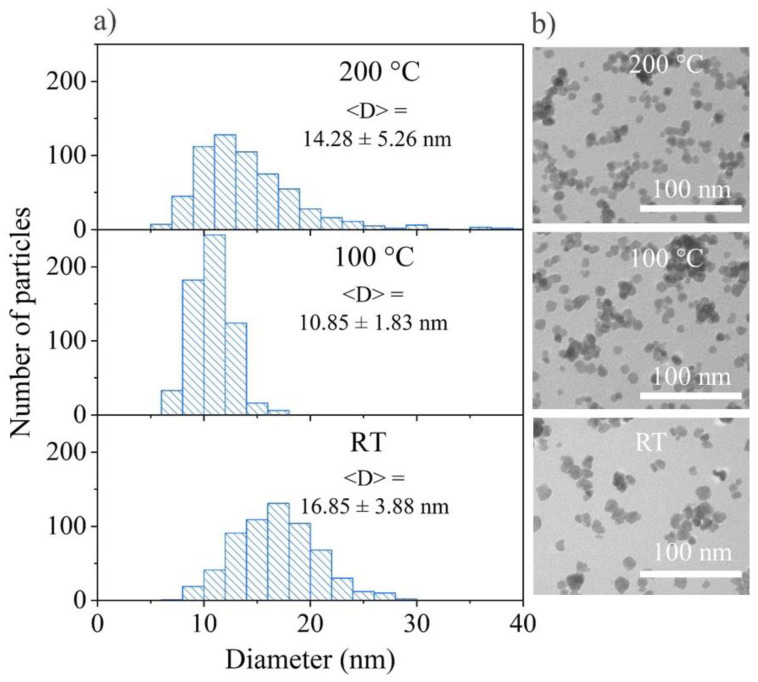
(**a**) Particle size distribution and <D> with the mean size ± standard deviation; (**b**) TEM images of 25Tb ST at room temperature, 100 °C, and 200 °C.

**Figure 9:**
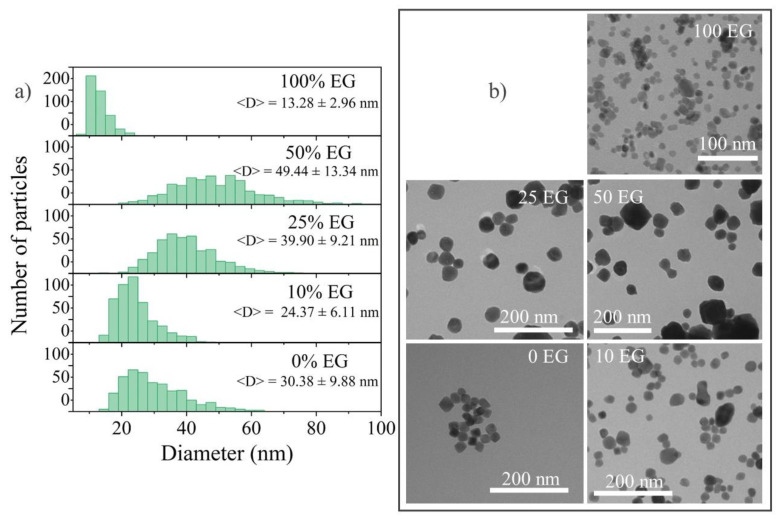
(**a**) Particle size distribution and <D> with the mean size ± standard deviation; (**b**) TEM images of 25Tb ST with different contents of EG 0, 10, 25, and 50%.

The shape, morphology, and size were also studied using transmission electron microscopy (TEM) for the nanoparticles synthesized by two different methods, MW and ST, in two ways: variation in the temperature and EG amount. As seen from the TEM images (Figure 6a,Figure 7a,Figure 8a,Figure 9a) and from the particle size distribution analysis (Figure 6b,Figure 7b,Figure 8b,Figure 9b), all of the 25Tb samples were characterized by the spherical shape of the particles.

The obtained averaged size of the particles (as estimated from TEM) is suitable for medical application. Indeed, such small nanoparticles are known to readily overcome biological barriers [33]. Small capillaries have a diameter of about 3 micrometers, and nanoparticles with a size less than 200 nm can be freely transported through the circulatory system and carry pharmaceutically active substances.

#### 3.2.3. Microwave Synthesis at Different Temperature and Different Percentage of EG

According to the particle size distribution, the sample synthesized at 200 °C had the most monodisperse character. This once again confirms that the standard procedure developed earlier provided the best result. Another two samples synthesized at 100 °C and 150 °C showed broader peaks and with increasing temperature, the peak shifted toward larger sizes. However, at 200 °C, we observed a peak shift to the small sizes. The average size of the sample synthesized at 100 °C was 8–9 nm, at 150 °C—10–11 nm, and at 200 °C—6–7 nm (Figure 6).

According to the particle size distribution of the sample synthesized with various shares of EG (Figure 7), almost no change was observed for 25Tb with 0, 10, 25, and 50% EG and the average size for these samples was 20–25 nm. In the case of adding 100% EG, the size of the nanoparticles sharply decreased to 6–8 nm. Similar results were obtained by the team of A. Opalinska for ZnO nanoparticles synthesized by microwave solvothermal synthesis with EG/water solvent [58]. Based on this research, the obtained NPs were characterized by a homogeneous morphology and a narrow distribution of particle sizes.

#### 3.2.4. Solvothermal Synthesis at Different Temperature and Different Percentage of EG

As evident from the particle size distribution, the samples synthesized at room temperature (RT) had an average size of 16–18 nm, at 100 °C it was 10–12 nm, and at 200 °C it was 11–13 nm (Figure 8).

It is easy to see from the particle size distribution of the samples synthesized with x% EG that the 0% EG particles had an average size of 22–25 nm, 10% EG—22–25 nm, 25% EG—34–43 nm, 50% EG—40–58 nm, and 100% EG—9–12 nm (Figure 9). As in the case of microwave syntheses, we observed a sharp decrease in the size of 100% EG nanoparticles as well as a narrower distribution of nanoparticles (i.e., a more monodisperse structure). On this basis, we can conclude that not only the type of heating affects a noticeable shift in the distribution, but also the type of solvent. The wider particle size distribution in the case of 25% EG and 50% EG may correspond to different rates of crystal nucleation and growth in different parts of the reaction space of the autoclave with a simultaneously high solution viscosity and an increase in the reaction rate upon the addition of water. An increase in the water content leads to a decrease in the viscosity and reaction rate. As a result, the difference in the rates of nucleation and the growth of crystallites decreases. Therefore, we could observe a decrease in the proportion of a large fraction of particles in 10% EG and 0% EG samples.

## 4. Conclusions

The BaGdF_5_ nanophosphors with different Tb:Gd ratios ranging from 0.1 to 0.5 were synthesized. Pure BaGdF_5_ phases were obtained. According to the XEOL analysis, the sample with the Tb:Gd ratio of 0.25 demonstrated the largest luminescence yield, being an optimal choice for converting X-rays into optical radiation.

Based on the optimal nanophosphor composition, the effect of temperature, the composition of EG/water solvent, and synthesis technique on the size of the obtained particles was next investigated for the samples with 25% of Tb ions. The microwave synthesis at all EG/water compositions makes it possible to obtain smaller particles with a narrower size distribution (7.29 ± 1.48 nm in 100%EG) compared to the solvothermal (13.3 ± 2.96 nm in 100% EG). This was due to the high heating rate and uniform mixture heating. The addition of 50% of water to ethylene glycol led to a sharp increase in the size of the resulting nanoparticles in both the MW and ST methods. Moreover, a further increase in the water content for MW synthesis had practically no effect on the size and width of the distribution of nanoparticles, but differences were noticeable in the case of solvothermal synthesis. For the sample synthesized in a mixture containing 50% EG, the largest nanoparticles with a wide size distribution (49.4 ± 13.3 nm) were observed, which was attributed to the different rates of nucleation and the growth of crystallites in different regions of the reaction volume. Increasing the water content led to a decrease in the nanoparticles’ size and a narrower distribution (24.3 ± 6.11 nm in 10%EG). This was due to an increase in the heating rate of the system due to an increase in the thermal conductivity of the solution and a decrease in its viscosity. Temperature variation in both the microwave and solvothermal syntheses did not lead to a noticeable change in the particle size.

Overall, as expected, MW heating produced smaller particles for all of the tested temperatures in comparison with the solvothermal synthesis. Thus, by varying the solvent composition as well as the method of system heating, we can achieve fine-tuning of the BaGdF_5_ nanoparticle sizes.

## Figures and Tables

**Table 1:** Amount of precursors used in the synthesis process.

Sample	BaCl_2_ ∙ 2H_2_O		GdCl_3_		TbCl_3_ ∙ 6H_2_O		NH_4_F	
	mg	mmol	mg	mmol	mg	mmol	mg	mmol
5Tb MW	244.3	1	250.4	0.95	13.3	0.05	203.7	5.5
10Tb MW			210.9	0.9	26.5	0.1		
25Tb MW			197.7	0.75	66.3	0.25		
50Tb MW			131.8	0.5	132.6	0.5		

**Table 2:** The synthesis conditions.

Type of Synthesis	Sample	% EG	Temperature, °C
Microwave (MW)	25Tb MW 100EG 200T	100	200
	25Tb MW 50EG	50	
	25Tb MW 25EG	25	
	25Tb MW 10EG	10	
	25Tb MW 0EG	0	
	25Tb MW 100EG 150T	100	150
	25Tb MW 100EG 100T	100	100
Solvothermal (ST)	25Tb ST 100EG 200T	100	200
	25Tb ST 50EG	50	
	25Tb ST 25EG	25	
	25Tb ST 10EG	10	
	25Tb ST 0EG	0	
	25Tb ST 100EG 100T	100	100
	25Tb ST 100EG RT	100	RT

**Table 3:** Cell parameters of the BaGdF_5_: x%Tb^3+^ samples calculated from the full profile analysis in the Jana2006 software and crystal size calculated using the Scherrer equation.

Sample	Expected Tb Content, at. %	Actual Tb Content, at. %	Cell Parameters. Å	Cell Volume. Å^3^	Goodness of Fit (GOF)	R-Factor	Crystal Size. nm
5Tb MW	0.71	0.45	5.9310(12)	208.64(7)	1.03	0.1282	8.53
10Tb MW	1.43	1.37	5.9279(14)	208.30(8)	1.12	0.1120	8.87
25Tb MW	3.57	3.78	5.9201(16)	207.49(3)	1.04	0.1546	7.67
50Tb MW	7.14	6.91	5.9034(9)	205.73(5)	0.99	0.1141	10.25

## Data Availability

All of the data are contained in the manuscript and its Supplementary Materials.

## Supplementary Materials

The following supporting information can be downloaded at: https://www.mdpi.com/article/10.3390/ma15238559/s1, Table S1: Calculated initial elemental composition of the BaGdF_5_:x%Tb^3+^ samples and the actual elemental composition from the X-ray fluorescence measurements; Figure S1: The diffraction profile and calculated line (upper pattern) and difference curve (lower pattern) of 10Tb (GOF = 1.12); Figure S2: The diffraction profile and calculated line (upper pattern) and difference curve (lower pattern) of 50Tb (GOF = 0.99).
