# Supplementary material for: Synthesis Optimization of BaGdF5:x%Tb3+ Nanophosphors for Tunable Particle Size
Source: Materials (Basel). 2022 Dec 1;15(23):8559. doi: 10.3390/ma15238559 (PMC9740830; doi:10.3390/ma15238559)
Supplement: Supplementary file 1 [file materials-15-08559-s001.zip › materials-2029683-supplementary.pdf]

materials
MDPI

Supplementary Materials: The following are available online at www.mdpi.com/xxx/s1,

- Table S1: Calculated initial elemental composition of the $BaGdF_5$:x%Tb^{3+} samples and actual elemental composition from X-ray fluorescence measurements,
- Figure S1: The diffraction profile and calculated line (upper pattern) and difference curve (lower pattern) of 10Tb (GOF = 1.12),
- Figure S2: The diffraction profile and calculated line (upper pattern) and difference curve (lower pattern) of 50Tb (GOF = 0.99).

Table S1. Calculated initial elemental composition of the $BaGdF_5$:x%Tb^{3+} samples and elemental composition from X-ray fluorescence measurements.

Sample names	Initial elemental composition at. %				Actual elemental composition at. %			
	Ba	Gd	Tb	F	Ba	Gd	Tb	F
0Tb	14.29	14.28	0	71.43	11.89	16.08	0	72.03
5Tb	14.29	13.57	0.71	71.43	9.1	17.72	0.45	72.73
10Tb	14.29	12.86	1.43	71.43	13.04	13.85	1.37	71.74
25Tb	14.29	10.71	3.57	71.43	13.09	11.40	3.78	71.73
50Tb	14.29	7.14	7.14	71.43	11.31	9.61	6.91	72.17

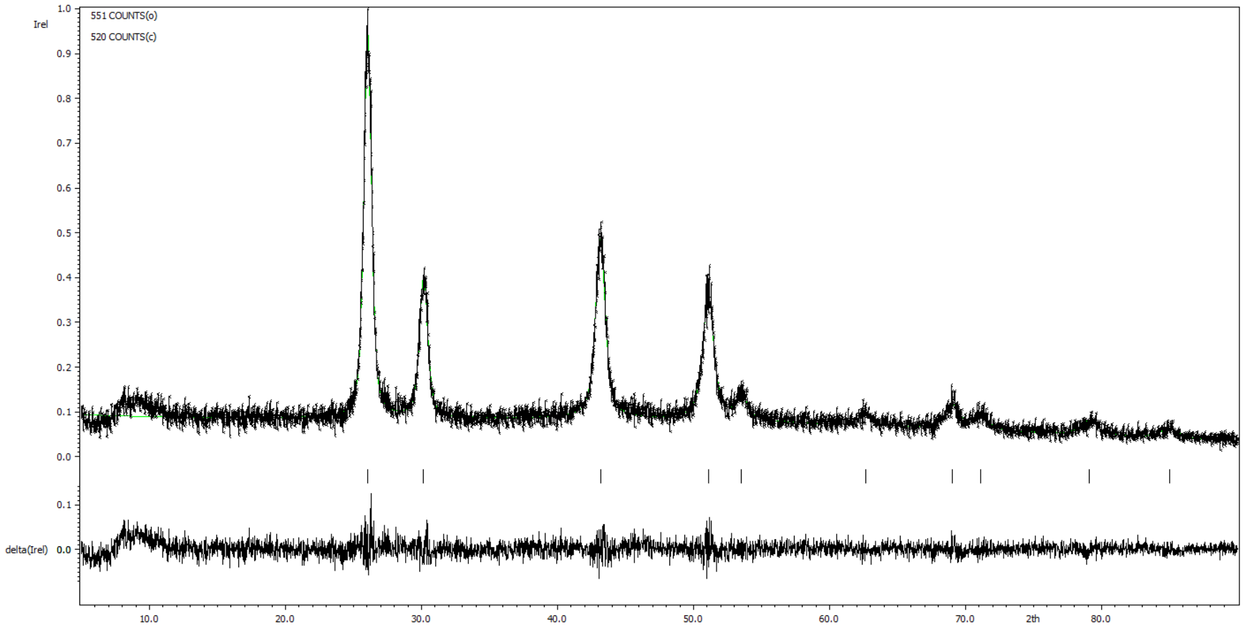

Figure S1. The diffraction profile and calculated line (upper pattern) and difference curve (lower pattern) of 10Tb (GOF = 1.12).

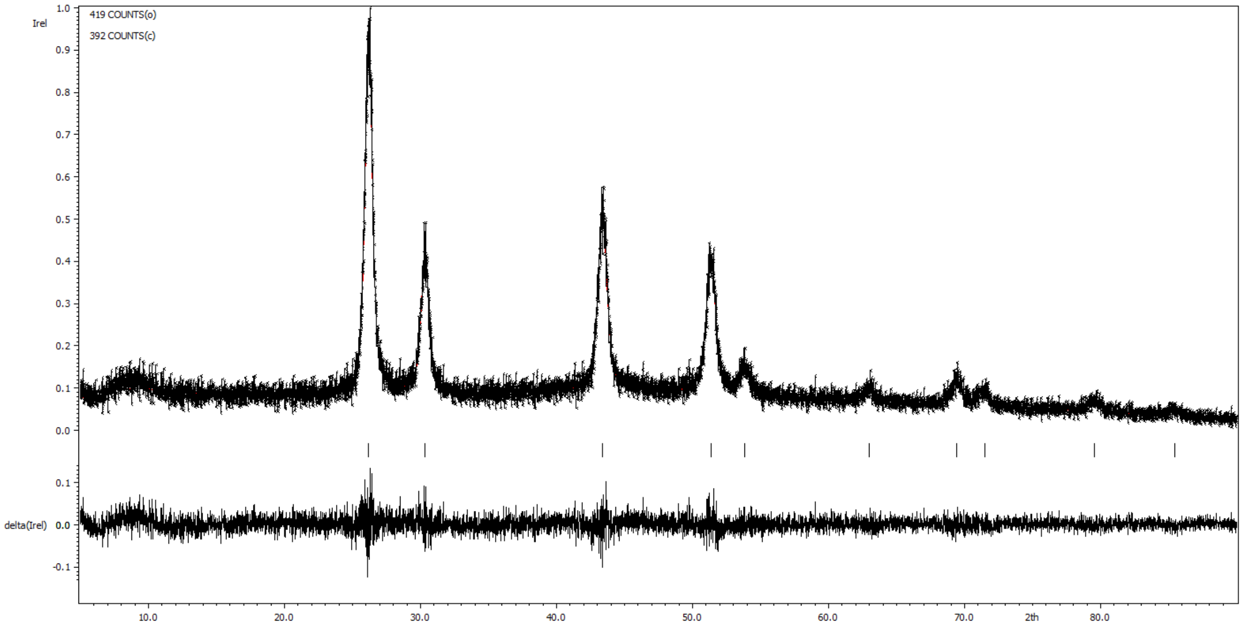

Figure S2. The diffraction profile and calculated line (upper pattern) and difference curve (lower pattern) of 50Tb (GOF = 0.99).
